# COVID-19 pandemic response in one of the world’s most complex and vulnerable settings

**DOI:** 10.1136/bmjgh-2022-009911

**Published:** 2022-06-27

**Authors:** Ziad A Memish, Richard John Brennan, Arash Rashidian, Abdinasir Abubakar, Wasiq Khan, Abdul Ghaffar

**Affiliations:** 1 Research & Innovation Center, King Saud Medical City, Riyadh, Saudi Arabia; 2 College of Mediicine, Alfaisal University, Riyadh, Saudi Arabia; 3 WHO Health Emergencies Programme, WHO Regional Office for the Eastern Mediterranean, Cairo, Egypt; 4 Science, Information and Dissemination, WHO Regional Office for the Eastern Mediterranean, Cairo, Egypt; 5 Alliance for Health Policy and Systems Research, World Health Organization, Geneva, Switzerland

**Keywords:** COVID-19, respiratory infections, SARS, public Health

Airborne diseases have plagued urban and rural communities alike in epidemic and pandemic forms since ancient times. During the 1918–1920 Spanish influenza pandemic, which was the deadliest in recorded history, it is estimated that more than 50 million people died.^1^ As of 1 May 2022, there have been over 510 million confirmed cases of SARS-CoV-2, a respiratory virus, and approximately 6.2 million deaths.^2^ Over the 2 years of pandemic, WHO has also estimated the excess mortality due to COVID-19, disease caused by SARS-CoV-2, to be 13.3–16.6 million deaths.^3^ Respiratory disease pandemics are clearly a threat to human security and development in our world.

The Eastern Mediterranean Region (EMR) is one of WHO’s six geographical regions and home to nearly 700 million people across 22 diverse countries and territories spread over South and West Asia, the Middle East and North Africa.^4^ A special issue series was commissioned in May 2021 jointly by the WHO Regional Office for the Eastern Mediterranean and *BMJ Global Health* to gather evidence on response and associated learnings from COVID-19 pandemic in the Region.^4^ This special issue explores the pandemic response with the aim of identifying successes, lessons and ways to address gaps for regional public health community to be able to better manage future pandemic risks.^5^ The Eastern Mediterranean Region is facing an unusual situation of multiple disease outbreaks ongoing in different countries (as shown in figure 1)^6^ along with the COVID-19 pandemic highlighting the importance of preparedness and response to epidemics and pandemics.

**Figure 1:**
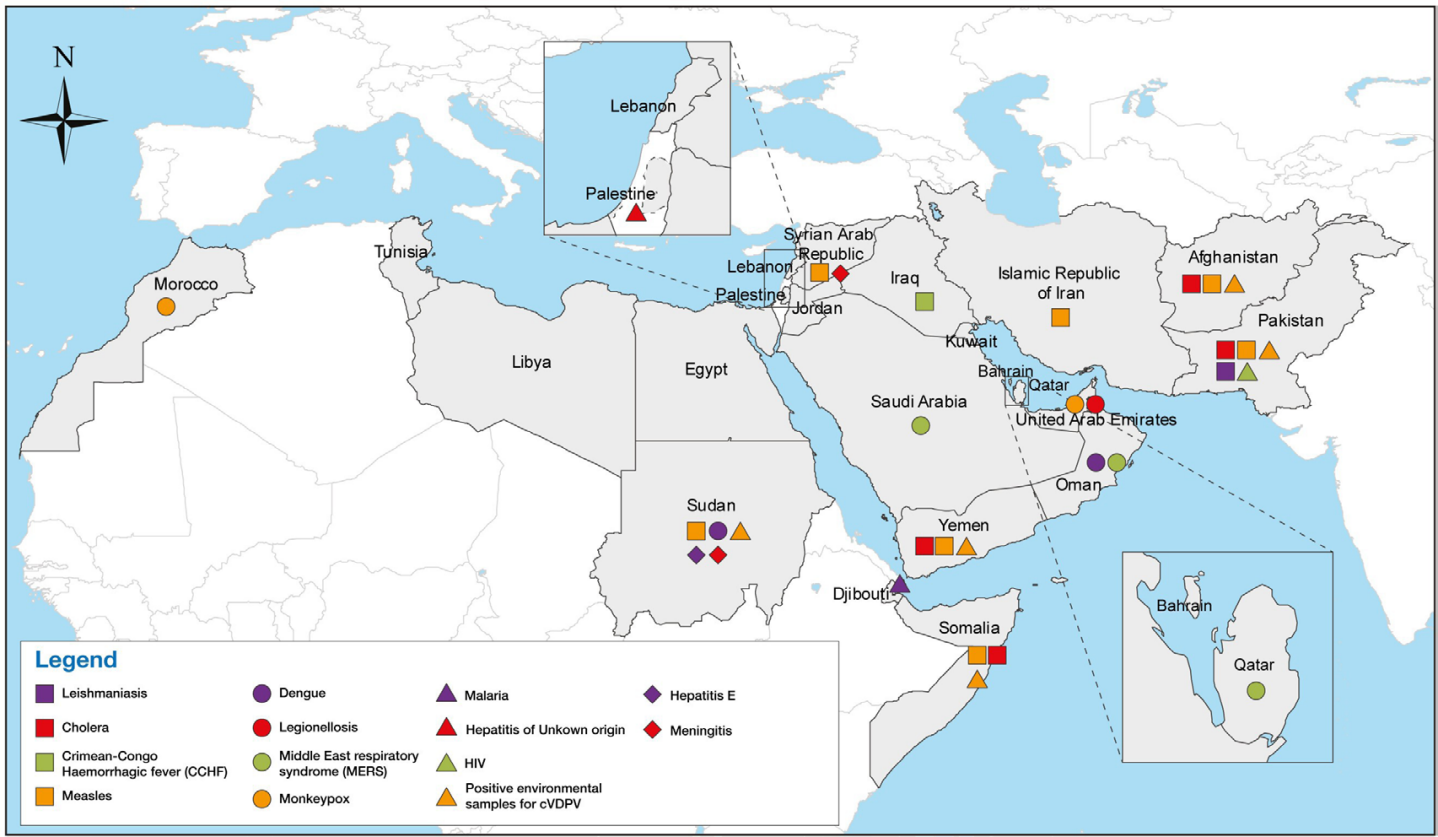
Disease outbreaks in WHO Eastern Mediterranean Region 2022.

The special issue begins by providing a summary of COVID-19 pandemic response efforts in the region and discussing the lessons learnt while working towards the end of the ongoing pandemic. The key message is that in the challenging context of Eastern Mediterranean countries, science combined with systems, societies and sense of solidarity can not only stall the impact of this pandemic but also pave the way for a resilient future.

The overview of pandemic response is followed by a practice paper investigating how resilient hospitals, as part of health systems, worked and can work better in complex environments amid the pandemic. To build resilience in health systems and mitigate the spread of infectious diseases of consequence,^7^ bottom-up and top-down approaches are instrumental to strengthen collaboration between and among policy-makers, public health officials, health facility managers, frontline healthcare workers and communities. In order to improve public health practice and policy, COVID-19 pandemic is reminding us that we must build intrinsically adaptive healthcare systems that respond to the actual needs of the people they serve. Evaluation of COVID-19 response during tertiary care hospitals in Iraq provides an example of a recovering yet fragile context to reinforce the significance of building systems’ resilience.

The pandemic has seen an explosion of research on COVID-19, although research unrelated to COVID-19 has generally been neglected as remote work modalities posed limitations.^8^ Kaiyue *et al* have discussed research and knowledge management on COVID-19 and how integrating an innovative research pillar approach into WHO’s Incident Management structure has been assisting countries to integrate evidence into strategic, operational and policy decisions. The research pillar addressed three areas, that is, (1) enhancing capacity for evidence generation at national level, (2) timely provision of available evidence to support pandemic response and (3) enhancing capacity and experience sharing in use of evidence. The authors have called for enhancing research that addresses social measures and behaviour change as most national research studies have remained focused on therapeutics.

COVID-19 has given a stark reminder that in crisis situations such as the pandemic caused by a previously unknown pathogen, repurposing existing knowledge, resources and technology through innovation is a necessity.^9^ In this issue, a case study from Oman on contact tracing describes the efforts of the Oman Ministry of Health to institute a digital contact tracing system to detect clusters of cases and enforce notification, testing and isolation. Interestingly, the number of contacted cases decreased as the pandemic progressed, with fewer cases over time with effective contact tracing efforts. Similar patterns have been reported in other countries, which may reflect a maturity in response (due to more efficiency in targeting, or a result of the way the pandemic has progressed in the country) or may have due to fatigue and continued pressure on the health systems as the pandemic was sustained over time. In a paper from Lebanon, Farah *et al* have presented a case where field testing was adopted to demonstrate that timely development and implementation of a testing strategy is significant during epidemic response. Authors though cautioned that testing alone will be insufficient if not complemented with contact tracing, risk communication and engagement with communities.

In the Eastern Mediterranean Region, low vaccine coverage has been attributed to limited supplies and weak immunisation systems. A case study from Djibouti presented in this issue shows how epidemiological data were used by the government to respond to a second surge in COVID-19 cases with deployment of vaccine. Since no one is safe until everyone is safe, international support is required to increase COVID-19 vaccination coverage, particularly in low-income and lower-middle-income countries.

As we move beyond the acute phase of the pandemic, it is likely that COVID-19 will attain endemic status. In a complex region such as the Eastern Mediterranean where conflict, frequent outbreaks, recurrent natural disasters and other vulnerabilities shape the daily lives of people and health systems, the COVID-19 pandemic has highlighted several lessons. Good planning and preparedness is key to responding quickly and effectively to pathogens of epidemic and pandemic potential, irrespective of the context, as it is for all emergencies. Contextual response needs more evidence and thus remedial strategies for example, in view of vital role of infection prevention and control in protecting human resources in already stressed and fragile environments. Global and regional solidarity for the equitable distribution of technical solutions (vaccines, diagnostics, therapeutics) is paramount for a speedy and effective response and recovery in pandemics, more so in compromised settings such as conflict zones. Responsible and decisive leadership at the highest levels of government is central to the effective control of COVID-19 pandemic, prevention of, preparedness for and response to the future pandemic threats.

## Data Availability

All data relevant to the study are included in the article or uploaded as supplementary information.
